# The Association and Predictive Value of Nutritional and Inflammatory Biomarkers in Advanced Non-Small Cell Lung Cancer Response to Immune Checkpoint Inhibitors

**DOI:** 10.3390/cancers18142185

**Published:** 2026-07-08

**Authors:** Mirte Dekker, Erick Suazo-Zepeda, T. Jeroen N. Hiltermann, Geertruida H. De Bock, Marjolein A. Heuvelmans

**Affiliations:** 1 Department of Epidemiology, University Medical Center Groningen, University of Groningen, 9713 GZ Groningen, The Netherlands; m.dekker03@umcg.nl (M.D.); g.h.de.bock@umcg.nl (G.H.D.B.); m.a.heuvelmans@umcg.nl (M.A.H.); 2 Department of Pulmonary, University Medical Center Groningen, University of Groningen, 9713 GZ Groningen, The Netherlands; t.j.n.hiltermann@umcg.nl; 3 Institute for Diagnostic Accuracy, 9713 GH Groningen, The Netherlands; 4 Department of Respiratory Medicine, Amsterdam University Medical Center, 1081 HV Amsterdam, The Netherlands

**Keywords:** carcinoma, non-small cell lung, biomarkers, disease progression, immune checkpoint inhibitors

## Abstract

**Background/Objectives**: Immune checkpoint inhibitors (ICIs) have broadened treatment options for non-small cell lung cancer (NSCLC), but many patients show limited response. Baseline biomarkers and indices, including C-reactive protein (CRP), albumin, Neutrophil-to-Lymphocyte Ratio (NLR), Glasgow Prognostic Score (GPS), Prognostic Nutrition Index (PNI), and Advanced Lung Cancer Inflammation Index (ALI), may predict treatment outcomes. This study evaluated whether these biomarkers associate with three-month mortality and disease progression assessed by disease control rate (DCR) using RECIST. **Methods**: We conducted a retrospective cohort study of consecutive patients with NSCLC from the OncoLifeS biobank (2015–2020). Three-month mortality was defined by status alive or deceased at three months after treatment start. DCR was based on CT-assessments using RECIST. Univariate and multivariable logistic regression models with backward selection and bootstrapping were developed to test biomarker associations with three-month mortality and DCR. Sensitivity analysis was performed to compare results with the standard definition of objective response rate (ORR). **Results**: Among 505 patients, 421 were alive and 84 deceased; 297 were responders and 208 non-responders. In the final progression model, higher GPS was associated with increased odds of 3-month progression, whereas higher ALI (OR 0.99, 95% CI 0.97–1.00) and higher PNI (OR 0.93, 95% CI 0.87–0.99) were associated with decreased odds of 3-month progression. Higher ALI (OR 0.97, 95% CI 0.94–0.99), and higher PNI (OR 0.83, 95% CI 0.78–0.89) were associated with lower odds of 3-month mortality. The mortality-model showed an AUC of 0.82 and 0.73 for the disease progression model. Sensitivity analysis with the standard RECIST definition revealed similar results. **Conclusions**: Higher GPS was associated with increased risk of progression, whereas higher PNI and ALI were associated with lower risk of progression and mortality. These findings warrant external validation before clinical implementation.

## Simple Summary

Patients with non-small cell lung cancer do not all equally benefit from immune checkpoint inhibitors, and there is a need for tools that can help with the identification of patients who are at higher risk of early disease progression and death. This study investigated whether commonly available blood-based biomarkers related to inflammation and nutritional status can predict outcomes in patients receiving immune checkpoint inhibitors. Data of these markers before treatment were collected from more than 500 patients and this data was compared with treatment response and mortality after treatment started. The findings suggest that higher levels of inflammation and poorer nutritional condition were associated with worse outcomes. These results help clinicians better estimate prognosis, support treatment decisions and improve personalized care for patients with non-small cell lung cancer.

## 1. Introduction

Lung cancer is the leading cause of cancer-related deaths, with non-small cell lung cancer (NSCLC) accounting for 85% of these cases [1,2]. Over the past decade, immune checkpoint inhibitors (ICIs) have revolutionized NSCLC treatment, significantly improving overall survival (OS) and progression-free survival (PFS) [3,4]. However, despite these advancements, the clinical benefits of ICIs remain highly variable, with significant differences in treatment response among patients [5,6].

Despite eligibility for ICI treatment, patients often face challenges like primary resistance and severe adverse effects undermining therapeutic success [7,8]. These challenges highlight the need for improved patient selection criteria. Additionally, a better understanding of the mechanisms underlying treatment variability is desired. Recent studies suggest that routinely measured nutritional and inflammatory biomarkers from blood tests may help predict treatment outcomes in oncology [9].

Several biomarkers, including neutrophil and lymphocyte counts, C-reactive protein (CRP), and albumin levels, have been associated with OS in cancer patients [10,11,12]. Moreover, composite indices derived from these biomarkers, such as the Neutrophil-to-Lymphocyte Ratio (NLR), Glasgow Prognostic Score (GPS), Prognostic Nutrition Index (PNI), and Advanced Lung Cancer Inflammation Index (ALI), have been linked to OS and PFS in NSCLC patients receiving ICIs [9]. Specifically, lower NLR, higher PNI, and higher ALI values have been associated with improved OS in NSCLC [13,14,15], while lower albumin levels, elevated CRP scores and higher GPS scores correlate with poorer survival outcomes [11,12,16].

Despite these findings, limited research has explored the relationship between these biomarkers and short-term mortality and disease progression.The predictive capabilities of baseline biomarkers might decline over time due to disease progression or the effect of treatment. It is relevant to evaluate short-term survival and treatment response in patients with advanced NSCLC treated with ICIs, as early identification of those at risk of death or progression can allow for timely treatment changes and potentially improve clinical outcomes, even when traditional predictors, such as PD-L1 expression, are not definitive [17]. Therefore, to contribute to the decision-making process regarding ICI treatment for lung cancer, this study aims to evaluate and internally validate the association between routinely measured biomarkers and composite indices with both three-month mortality and treatment response assessed using a modified disease control rate (DCR) definition based on RECIST categories, in which stable disease lasting ≥6 months was classified as disease control.

## 2. Materials and Methods

### 2.1. Study Design and Participants

We performed a retrospective cohort study using real-world data from the data bio-bank Oncological Life Study: Living well as a cancer survivor (OncoLifeS) from the University Medical Center Groningen (UMCG). This biobank connects biological samples with clinical and patient-reported outcomes, allowing for a thorough understanding of cancer survivorship [18]. Ethical approval for OncoLifeS has been granted by the Medical Ethics Committee of the UMCG (no. 2010/109). All patients in this databiobank signed written informed consent.

We included patients aged 18 years or older who were diagnosed with stage III or IV NSCLC and received at least one cycle of ICIs, either as monotherapy or in combination with chemotherapy or other ICIs. Eligible patients had biomarker data, including serum albumin, CRP levels, neutrophil count, and lymphocyte count, collected no more than two weeks before the start of ICI treatment. The ICIs prescribed included Pembrolizumab, Nivolumab, Atezolizumab, Avelumab, Durvalumab, Ipilimumab, and Tremelimumab.

### 2.2. Data Collection

Clinical data were collected from electronic medical records before the start of treatment, including patients’ sex, age, BMI, clinical stage, line of treatment, mono- and combination therapy, comorbidities, such as diabetes, hypertension, COPD, rheumatological conditions, dementia and cardiovascular diseases, and the biomarker values on serum albumin, CRP level, neutrophil- and lymphocyte count. These biomarkers were used to calculate the indices NLR, GPS, ALI and PNI according to previous literature protocols (Table S1). The evaluation of the three and six-month treatment response was also extracted from medical records. The date of death was obtained from the Dutch Personal Records Database (BRP).

### 2.3. Outcomes

The two primary outcomes of this study were three-month mortality and DCR as defined by RECIST. Three-month mortality was defined as death by any cause three months after treatment initiation. DCR was determined by CT-assessed treatment response to ICI therapy, evaluated using RECIST criteria by lung specialists three or six months after the start of treatment. RECIST includes the following categories: (1) complete response (CR), (2) partial response (PR), (3) stable disease (SD), and (4) progressive disease (PD) [19]. For the three-month mortality analysis, patients were categorized as alive or deceased. For the DCR analysis, patients were classified into two groups: responders, defined as those who had a CR at 3 months after the start of treatment, PR at 3 months after the start of treatment, SD ≥ 6 months after the start of treatment, and non-responders, being patients with SD < 6 months, PD within 3 months after the start of treatment or death within 3 months after the start of treatment. This definition is a modification of the standard RECIST-based objective response rate (ORR), which only includes CR and PR. This approach is chosen since it better reflects clinically relevant disease control in the context of a short-term primary endpoint.

### 2.4. Statistical Analysis

#### 2.4.1. Descriptive Statistics, Comparison Biomarkers Between Treatment Lines and Univariate Analysis

Descriptive statistics were reported as N (%) or as mean and standard deviations. Univariate logistic regression analysis were performed to assess the association between individual biomarkers and three-month mortality and disease progression. Complete-case analyses were performed. In addition, baseline biomarker values between treatment lines were assessed with Kruskal-Wallis test for continuous variables and chi-square test for categorical variables.

#### 2.4.2. Multivariable Model Development and Variable Selection

Separate multivariable logistic regression models were developed for 3-month progression and 3-month mortality. Candidate biomarker predictors were GPS, ALI, PNI, and NLR. Clinical covariates (age, sex, TNM stage, monotherapy, treatment line, and comorbidity status) were included a priori and retained in all the adjusted models irrespective of statistical significance.

Model selection was performed by sequentially removing biomarkers with limited contribution to model performance. Variable retention was evaluated using likelihood-ratio tests, Akaike Information Criterion (AIC), and changes in discrimination. Model discrimination was assessed using the area under the receiver operating characteristic curve (AUC), and AUCs were compared using DeLong’s test.

Multicollinearity was assessed using the generalized variance inflation factor (GVIF), with values > 5 considered indicative of problematic collinearity. Complete-case analyses were performed.

#### 2.4.3. Bootstrap

Internal validation was conducted using 1000 bootstrap resamples. Optimism-corrected AUCs were calculated by subtracting the average optimism from the apparent AUC. Bootstrap distributions were used to evaluate the stability of regression coefficients and derive bootstrap-based 95% confidence intervals.

#### 2.4.4. Sensitivity Analysis

A sensitivity analysis was performed using a standard RECIST-based 3-month progression endpoint (ORR). The same multivariable logistic regression model used in the primary analysis was fitted, including GPS, ALI, and PNI, adjusted for age, sex, clinical stage, treatment regimen, treatment line, and comorbidities. Model discrimination was assessed using the area under the AUC.

IBM SPSS version 28 and R version 4.5.1 were used to perform statistical analysis.

## 3. Results

### 3.1. Descriptive Statistics

We included 505 adult patients with NSCLC who were treated with ICI. In our cohort, 421 patients (83.4%) were alive, while 84 patients (16.6%) had died. Additionally, disease progression was assessed, indicating that 297 (58.8%) were classified as responders, while 208 (41.2%) experienced disease progression or died before the three-month response or stable disease assessment, which required at least a 6-month follow-up.

In this study population, most patients were male (N = 307, 60.8%), and the majority were diagnosed with stage IV NSCLC (N = 445, 88.1%). On average, patients had an age of 64.7 years (SD = 9.3) and a mean BMI of 25.8 (SD = 4.4). A considerable proportion of patients had comorbidities (N = 352, 69.7%) and were primarily treated with ICIs (N = 423, 83.8%) (Table 1).

Several baseline biomarkers differed significantly between treatment lines, including CRP, albumin, GPS and PNI. CRP values were highest in patient receiving third-line treatment. PNI levels were similar in treatment lines 2 and 3, 40.0 (38.0–43.0) vs. 40.0 (36.0–42.0), whereas slightly higher values were observed in first-line treatment: 41.0 (38.0–44.0). Between all treatment lines, GPS 1 was the most frequently observed category (50.6% vs. 57.8% vs. 59.2%) (Table S2).

### 3.2. Distribution of Inflammatory and Nutritional Biomarkers and Their Univariate Associations with Three-Month Mortality and Disease Progression

The distribution of inflammatory and nutritional biomarkers was similar for three-month mortality and DCR. Responders and survivors generally exhibit lower levels of inflammatory markers and higher levels of nutritional biomarkers.

CRP levels displayed a comparable pattern, with lower levels observed in survivors (33.6, SD = 43.8) compared to deceased patients (95.5, SD = 76.7). Responders also showed lower CRP levels (29.2, SD = 39.9) than non-responders (65.3, SD = 67.6). In univariate analysis, higher CRP was significantly associated with three-month mortality and decreased odds of achieving an DCR (OR for mortality = 1.02; 95% CI: 1.01–1.02, OR for DCR = 1.01; 95% CI: 1.01–1.02).

NLR was elevated in deceased patients (8.9, SD = 8.6) compared to survivors (5.7, SD = 4.4), and a similar distribution was noticed in non-responders (7.2, SD = 6.6) compared to responders (5.6, SD = 4.5). A higher NLR was significantly associated with three-month mortality and decreased odds of achieving an DCR (OR for mortality = 1.09; 95% CI: 1.05–1.14, OR for DCR = 1.06; 95% CI: 1.02–1.10).

In contrast to inflammatory markers, nutritional biomarkers, such as PNI and ALI, were higher in survivors and responders. The mean albumin level was higher in survivors (40.7, SD = 4.2) compared to deceased patients (36.0, SD = 4.8), and a similar pattern was seen in responders (41.1, SD = 3.9) compared to non-responders (38.2, SD = 5.0). Lower albumin levels were associated with three-month mortality and decreased odds of achieving an DCR (OR for mortality = 0.81; 95% CI: 0.77–0.86, OR for DCR = 0.86; 95% CI: 0.82–0.90).

The mean PNI was higher among survivors (40.8, SD = 4.0) and responders (41.2, SD = 3.8) than among deceased patients (36.0, SD = 4.9) and non-responders (38.4, SD = 4.9). Univariate analysis indicated that lower PNI values were significantly associated with mortality and worse treatment response (OR for mortality = 0.80; 95% CI: 0.75–0.84, OR for DCR = 0.86; 95% CI: 0.82–0.90).

ALI followed the same distribution, with higher values in the survivors (29.0, SD = 23.6) and responders group (30.7, SD = 25.7) compared to deceased patients (15.9, SD = 10.4) and non-responder group (21.4, SD = 15.4). Decreased ALI values showed a significant association with three-month mortality and decreased odds of achieving an DCR (OR for mortality = 0.94; 95% CI: 0.91–0.96, OR for DCR = 0.97; 95% CI: 0.96–0.99).

Finally, GPS scores displayed similar distributions across mortality and DCR analyses. The majority of patients had a GPS score of 1, with comparable proportions observed among survivors (N = 222, 55.2%) and deceased patients (N = 47, 56.6%). A GPS score of 1 (OR = 4.66; 95% CI: 2.05–10.85) and a GPS score of 2 (OR = 24.54; 95% CI: 9.74–61.83) were significantly associated with three-month mortality. Regarding treatment response, 143 responders (50.4%) and 126 non-responders (62.7%) had a GPS score of 1. Higher GPS scores were significantly associated with DCR, with a score of 1 (OR = 3.17; 95% CI: 2.03–4.95) and a GPS score of 2 (OR = 9.60; 95% CI: 4.76–19.37) associated with poorer treatment outcomes (Table 2 and Table 3).

### 3.3. Model Development and Performance

A total of 460 patients with complete data were included in the three-month mortality analysis, of whom 75 (16.3%) died within three months after treatment initiation. For the 3-month progression analysis, 452 patients with complete data were included, of whom 184 (40.7%) progressed within three months after treatment initiation.

#### 3.3.1. Three-Month Mortality

In the adjusted model including all candidate biomarkers, higher ALI (OR 0.96, 95% CI 0.93–0.99) and higher PNI (OR 0.88, 95% CI 0.80–0.96) were associated with lower odds of 3-month mortality. GPS (OR 1.82, 95% CI 0.91–3.81) and NLR (OR 0.98, 95% CI 0.93–1.04) did not demonstrate independent associations with 3-month mortality (Table 4).

Sequential model reduction demonstrated that removal of GPS and NLR resulted in a more parsimonious model with minimal changes in model fit and discrimination. The full adjusted model achieved an AUC of 0.824, compared with an AUC of 0.817 for the final parsimonious model. Model fit remained comparable after exclusion of GPS and NLR, supporting selection of the simpler model.

In the final mortality model, higher ALI remained associated with lower odds of 3-month mortality (OR 0.97, 95% CI 0.94–0.99), and higher PNI was strongly associated with lower odds of 3-month mortality (OR 0.83, 95% CI 0.78–0.89). The final model demonstrated good discriminatory performance, with an AUC of 0.817 (Figure S1).

#### 3.3.2. Three-Month Progression

In the adjusted model including all candidate biomarkers, higher GPS was associated with increased odds of 3-month progression (OR 1.75, 95% CI 1.10–2.82), whereas higher ALI (OR 0.98, 95% CI 0.97–1.00) and higher PNI (OR 0.93, 95% CI 0.87–0.99) were associated with lower odds of 3-month progression. NLR was not associated with 3-month progression (OR 0.99, 95% CI 0.94–1.04) (Table 4).

Because NLR did not improve model fit or discrimination, it was removed from the final model. Model fit improved slightly following exclusion of NLR (AIC 555.8 vs. 557.6), while discrimination remained virtually unchanged (AUC 0.735 vs. 0.736).

In the final progression model, higher GPS remained associated with increased odds of 3-month progression (OR 1.75, 95% CI 1.10–2.81), whereas higher ALI (OR 0.99, 95% CI 0.97–1.00) and higher PNI (OR 0.93, 95% CI 0.87–0.99) remained associated with lower odds of 3-month progression. Among the clinical covariates, advanced stage disease was associated with increased odds of 3-month progression (OR 2.15, 95% CI 1.07–4.56), as was treatment with monotherapy (OR 2.29, 95% CI 1.23–4.43) (Table 4). The final progression model showed acceptable discriminatory performance, with an AUC of 0.735 (Figure S2).

#### 3.3.3. Multicollinearity

No evidence of problematic multicollinearity was observed in either final model. Adjusted generalized variance inflation factor (GVIF^(1/(2 × Df))) values ranged from 1.03 to 1.32 in the final 3-month progression model and from 1.03 to 1.11 in the final 3-month mortality model, indicating minimal correlation among predictors and stable coefficient estimates (Table S3).

#### 3.3.4. Bootstrap

Internal validation using 1000 bootstrap samples demonstrated good stability of the final models.For the 3-month mortality model, the apparent AUC was 0.817 and the optimism-corrected AUC was 0.791. The bootstrap analysis confirmed the stability of the biomarker effects, with lower ALI (OR 0.96, 95% CI 0.94–0.99) and lower PNI (OR 0.83, 95% CI 0.76–0.89) remaining independently associated with increased odds of 3-month mortality (Table 5). 

For the 3-month progression model, the apparent AUC was 0.735 and the optimism-corrected AUC was 0.711, indicating limited overfitting. The bootstrap estimates confirmed the associations observed in the original model, with higher GPS (OR 1.79, 95% CI 1.10–2.99), lower ALI (OR 0.99, 95% CI 0.97–1.00), and lower PNI (OR 0.92, 95% CI 0.86–0.99) remaining associated with increased odds of 3-month progression (Table 5).No substantial changes in the direction or magnitude of the regression coefficients were observed across bootstrap samples.

#### 3.3.5. Sensitivity Analysis

Using the RECIST-based endpoint (ORR), only 4 of 505 patients (0.8%) were reclassified compared with the primary analysis. Results were comparable to the primary model. Higher GPS was associated with increased odds of 3-month progression (OR 1.67, 95% CI 1.05–2.66), whereas higher ALI(OR 0.99, 95% CI 0.97–1.00) and PNI (OR 0.93, 95% CI 0.87–0.99) were associated with lower odds of 3-month progression (Table S4). Model discrimination was similar to the primary analysis (AUC 0.732 vs. 0.729) (Figure S3).

## 4. Discussion

### 4.1. Key Results

In this retrospective real-world cohort study, we evaluated the association between baseline biomarkers (albumin, CRP, NLR, GPS, PNI, and ALI) and the clinical adjustment variables (age, sex, TNM stage, monotherapy, treatment line, and comorbidity status) with both three-month mortality and DCR as defined by RECIST, with incorporation of stable disease ≥ 6 months in the responder group, in patients with NSCLC treated with ICIs. A higher GPS, lower PNI and ALI were associated with three-month disease progression, whereas only a lower PNI and ALI were associated with three-month mortality. Both models demonstrated good discriminatory performance, with an AUC of 0.82 for mortality and 0.74 for disease progression. Sensitivity analysis showed consistent results with the primary model.

### 4.2. Interpretation

Our findings align with previous studies indicating that higher GPS scores have been associated with poorer outcomes, whereas higher PNI and ALI have been associated with better outcomes [12,14,15,16]. This relationship may be explained by the role of systemic inflammation in cancer progression, as a less inflammatory tumor microenvironment is often linked to improved outcomes [20]. CRP, which is a component of GPS, may not only serve as a marker of systemic inflammation but could also play a more direct role in immune modulation. CRP has been shown to activate the classical complement pathway and to interact with Fc receptors on immune cells, which could lead to modulation of inflammatory and immune receptors [21]. Complement activation has been associated with the development of an immunosuppressive tumor microenvironment as well, which potentially impairs an effective anti-tumor response and therefore reduces the efficacy of ICI treatment [22]. CRP showed strong associations with both progression and mortality in the univariable analyses. However, CRP was not retained in the final multivariable models because its prognostic information overlapped with the composite biomarkers.

Similarly, hypoalbuminemia, a key component of GPS and PNI, reflects a systemic inflammatory state, since pro-inflammatory cytokines such as IL-6 suppress hepatic albumin synthesis [23]. In addition, albumin may influence drug distribution and pharmacokinetics, as it plays a key role in drug binding and transport. Lower albumin levels have been associated with increased clearance of therapeutic antibodies, potentially reducing exposure to ICI therapy [24]. This may partly explain why a higher GPS, which indicates a lower albumin level, is associated with poor prognosis [16]. In our study, GPS was only associated with disease progression. This marker may be more directly related to tumor progression than overall survival, since it focuses on systemic inflammation solely. However, the estimate for GPS 2 should be interpreted with caution, as the small subgroup size resulted in greater uncertainty, which is reflected by the wide CI.A lower PNI, however, was associated with both three-month mortality and disease progression. This marker reflects not only systemic inflammation and nutritional status, but also incorporates immune competence, by including lymphocyte count, which may be particularly relevant for treatment with ICIs.

Although NLR has been previously reported as a predictor of better outcomes in patients with NSCLC treated with first-line pembrolizumab, in the presence of PD-L1 TPS ≥50% and without EGFR or ALK genomic alterations [13], it was not a significant predictor in our study. These prior findings may reflect a more favorable and biologically homogenous patient population. Moreover, this discrepancy may be due to the inclusion of ALI in our model, as ALI incorporates NLR and may serve as a more comprehensive predictor of early progression.

Despite showing moderate discrimination (AUC of 0.82 for mortality and 0.73 for progression), the model is not yet suitable as a clinical decision tool since no externally validated risk thresholds were defined to guide treatment decisions.

### 4.3. Strengths and Limitations

This study has several strengths. The consecutive inclusion of patients with NSCLC from the OncoLifeS dataset and the availability of comprehensive pre-treatment biomarker data enhance the robustness of our findings. This study focused on short-term outcomes, namely three-month mortality and treatment response after initiation of ICI treatment. We used a DCR that included patients with stable disease lasting at least six months, as well as those achieving PR or CR. This differs from the conventional definition of ORR, which includes only PR and CR. This approach was chosen because durable disease stabilization was considered as a clinically meaningful outcome in our study population, given that the median survival of patients with metastatic NSCLC ranges from 3–34 months [25]. Importantly, a sensitivity analysis using the standard definition of ORR was also performed and yielded consistent results, supporting the robustness of our findings.

However, some limitations should be acknowledged. This study focused on a short-term endpoint of three-month mortality and treatment response after initiation of treatment. By using a fixed three-month endpoint, patients who experienced progression or death after this time point were classified as non-events. Consequently, our analyses did not entail time-to-event data on disease progression or survival, and did not allow for evaluation of the evolvement of the predictive value from the baseline biomarkers. Therefore, the findings should be interpreted as associations with short-term outcomes rather than long-term prognosis. 

Moreover, the lack of cause-specific mortality data implies that different causes of death, including disease progression, treatment-related toxicity and comorbidities could not be distinguished. Treatment-related toxicity could also indirectly reflect treatment response, and could therefore contain clinically relevant information about prognosis. Also, due to limited availability of detailed data on comorbidities, individual conditions such as COPD and rheumatological disease could not be modelled separately and were therefore combined in one single binary comorbidity, which may have masked condition specific effects on inflammatory markers and outcomes.

As the UMCG is a tertiary referral hospital, its patient population predominantly consists of individuals with advanced disease. Consequently, our cohort may not be fully representative of patients treated in secondary care settings. This may have led to an overestimation of the predictive performance of the nutritional and inflammatory markers, thereby limiting the generalizability of our findings to patients with a more favorable prognosis. However, during the early implementation phase of ICIs (2015–2018), the treatment was centralized at the UMCG. As a result, patients treated during this period likely reflect a broader real-world population, whereas those treated between 2019–2021 may represent a more selected and clinically complex subgroup.

Due to the study period, PD-L1 expression was unavailable in 161 of 505 patients (31.9%), as PD-L1 testing was not routinely performed. Currently, PD-L1 is the most important biomarker in ICI treatment, and exclusion of this biomarker in our study could have influenced the completeness of available predictive information. Also, ICI strategies have evolved substantially, including increased use of combination regimens and changes in patient selection. Therefore, while our findings support the prognostic value of baseline inflammatory and nutritional biomarkers, their direct generalizability to current ICI treatment regimens should be interpreted with caution.

Another limitation of the study is its retrospective study design. The available sample size and number of events may have limited the complexity of the models that could be reliably developed. Although internal validation indicated that the models performed consistently within our population, external validation is necessary to determine their generalizability to independent patient cohorts.

## 5. Conclusions

Baseline biomarkers routinely measured in standard blood draws, including PNI and ALI, were associated with three-month mortality. In the final multivariable model, lower PNI and lower ALI remained independently predictive of mortality in our cohort of patients with NSCLC treated with ICIs. Similarly, higher GPS, lower PNI and lower ALI were predictive of disease progression. These associations may reflect the influence of systemic inflammation and general nutritional status on cancer progression, both of which can affect survival and treatment response. In particular, biomarkers indicative of a less inflammatory tumor microenvironment, such as lower GPS, and a better nutritional status, such as higher PNI and ALI, are linked to more favorable outcomes. These findings suggest that inflammatory and nutritional biomarkers may contribute to risk stratification in patients treated with ICIs. However, these findings should be externally validated in independent patient cohorts before implementation in routine clinical practice can be considered.

## Figures and Tables

**Table 1:** Demographic and Clinical Characteristics of Patients (N = 505), stratified by Three-Month Mortality and Disease Progression in Patients with Non-Small Cell Lung Cancer Treated with Immune Checkpoint Inhibitors (Alive and Deceased/Responders and Non-responders).

		Three-Month Mortality		DCR	
		Alive N = 421	Deceased N = 84	Responders N = 297	Non-Responders N = 208
		N (%)	N (%)	N (%)	N (%)
Sex	Male	254 (60.3)	53 (63.1)	180 (60.6)	127 (61.1)
	Female	167 (39.7)	31 (36.9)	117 (39.4)	81 (38.9)
Mean Age, years (SD)		64.7 (9.2)	64.9 (9.5)	64.8 (9.4)	64.7 (9.2)
Mean BMI, kg/m^2^ (SD)		26.0 (4.3)	24.8 (4.4)	26.3 (4.4)	25.1 (4.2)
	Missing	22 (5.2)	3 (3.6)	14 (4.7)	11 (5.3)
Clinical stage	III	53 (12.6)	7 (8.3)	44 (14.8)	16 (7.7)
	IV	368 (87.4)	77 (91.7)	253 (85.2)	192 (92.3)
Comorbidities ^a^	Yes	293 (69.6)	59 (70.2)	211 (71.0)	141 (67.8)
	No	128 (30.4)	25 (29.8)	86 (29.0)	67 (32.2)
Monotherapy	Yes	347 (82.4)	76 (90.5)	234 (78.8)	189 (90.9)
	No	74 (17.6)	8 (9.5)	63 (21.2)	19 (9.1)
Treatment line	First Line	154 (37%)	23 (27%)	125 (42%)	52 (25%)
	Second line	229 (54%)	47 (56%)	147 (49%)	129 (63%)
	Third and further line	38 (9.0%)	14 (17%)	29 (9.6%)	23 (11%)

Abbreviations: BMI = Body Mass Index. ^a^. Included diabetes, hypertension, COPD, rheumatological conditions, dementia and cardiovascular diseases.

**Table 2:** Nutritional and Inflammatory Biomarker and Indexes of Patients (N = 505), Stratified by Three-Month Mortality (Alive and Deceased). Univariate Analyses of Nutritional and Inflammatory Biomarkers and Indexes Associated with Three-Month Mortality in Patients with Non-Small Cell Lung Cancer Treated with Immune Checkpoint Inhibitors.

		Alive N = 421		Deceased N = 84		Univariate Analysis			
		N (%)	Mean (SD)	N (%)	Mean (SD)	OR	95% LCI	95% UCI	*p*-Value
Albumin			40.7 (4.2)		36.0 (4.8)	0.81	0.77	0.86	<0.001
	Missing	15 (3.6)		0 (0.0)					
CRP			33.6 (43.8)		95.5 (76.7)	1.02	1.01	1.02	<0.001
	Missing	19 (4.5)		1 (1.2)					
NLR			5.7 (4.4)		8.9 (8.6)	1.09	1.05	1.14	<0.001
	Missing	24 (5.7)		5 (6.0)					
GPS	0	154 (38.3)		7 (8.4)					
	1	222 (55.2)		47 (56.6)		4.66	2.05	10.58	<0.001
	2	26 (6.5)		29 (34.9)		24.54	9.74	61.83	<0.001
	Missing	19 (4.5)		1 (1.2)					
PNI			40.8 (4.0)		36.0 (4.9)	0.80	0.75	0.84	<0.001
	Missing	22 (5.2)		4 (4.8)					
ALI			29.0 (23.6)		15.9 (10.4)	0.94	0.91	0.96	<0.001
	Missing	33 (7.8)		8 (9.5)					

Abbreviations: CRP = C-Reactive Protein (mg/L); NLR = Neutrophil to Lymphocyte Ratio (absolute count × 10^9^/L); GPS = Glasgow Prognostic Score, Glasgow Prognostic Score 0 = CRP ≤ 10 (mg/L) and albumin ≥ 35 (g/L), Glasgow Prognostic Score 1 = CRP > 10 (mg/L) and albumin ≥ 35 (g/L) or CRP ≤ 10 (mg/L) and albumin < 35 (g/L), Glasgow Prognostic Score 2 = CRP > 10 (mg/L) and albumin < 35 (g/L); PNI = Prognostic Nutrition Index = 10 × serum albumin value (g/dL) + 0.005 × total lymphocyte count/mm^3^; ALI = Advanced Lung Cancer Inflammation Index = BMI (kg/m^2^) × albumin (g/dL)/NLR; BMI = Body Mass Index.

**Table 3:** Nutritional and Inflammatory Biomarker and Indexes of Patients (N = 505), Stratified by Disease Progression (Responders and Non-responders). Univariate Analyses of Nutritional and Inflammatory Biomarkers and Indexes Associated with Disease Progression in Patients with Non-Small Cell Lung Cancer Treated with Immune Checkpoint Inhibitors.

		Responders N = 297		Non-Responders N = 208		Univariate Analysis			
		N (%)	Mean (SD)	N (%)	Mean (SD)	OR	95% LCI	95% UCI	*p*-Value
Albumin			41.1 (3.9)		38.2 (5.0)	0.86	0.82	0.90	<0.001
	Missing	10 (3.4)		5 (2.4)					
CRP			29.2 (39.9)		65.3 (67.6)	1.01	1.01	1.02	<0.001
	Missing	13 (4.4)		7 (3.4)					
NLR			5.6 (4.4)		7.2 (6.6)	1.06	1.02	1.10	<0.001
	Missing	16 (5.4)		13 (6.3)					
GPS	0	126 (44.4)		35 (17.4)					
	1	143 (50.4)		126 (62.7)		3.17	2.03	4.95	<0.001
	2	15 (5.3)		40 (19.9)		9.60	4.76	19.37	<0.001
	Missing	13 (4.6)		7 (3.4)					
PNI			41.2 (3.8)		38.4 (4.9)	0.86	0.82	0.90	<0.001
	Missing	14 (4.7)		12 (5.8)					
ALI			30.7 (25.7)		21.4 (15.4)	0.97	0.96	0.99	<0.001
	Missing	22 (7.4)		19 (9.1)					

Abbreviations: CRP = C-Reactive Protein (mg/L); NLR = Neutrophil to Lymphocyte Ratio (absolute count × 10^9^/L); GPS = Glasgow Prognostic Score, Glasgow Prognostic Score 0 = CRP ≤ 10 (mg/L) and albumin ≥ 35 (g/L), Glasgow Prognostic Score 1 = CRP > 10 (mg/L) and albumin ≥ 35 (g/L) or CRP ≤ 10 (mg/L) and albumin < 35 (g/L), Glasgow Prognostic Score 2 = CRP > 10 (mg/L) and albumin < 35 (g/L); PNI = Prognostic Nutrition Index = 10 × serum albumin value (g/dL) + 0.005 × total lymphocyte count/mm^3^; ALI = Advanced Lung Cancer Inflammation Index = BMI (kg/m^2^) × albumin (g/dL)/NLR; BMI = Body Mass Index.

**Table 4:** Multivariable Logistic Regression Model (Backward Selection) of Nutritional and Inflammatory Biomarkers and Indexes Associated with Three-Month Mortality and Disease Progression in Patients with Non-Small Cell Lung Cancer Treated with Immune Checkpoint Inhibitors.

		Multivariable Regression Model (All Candidate Variables Included)				Multivariable Regression Model Final Model			
Variable		OR	95% LCI	95% UCI	*p*-Value	OR	95% LCI	95% UCI	*p*-Value
NLR	Three-month mortality	0.98	0.93	1.04	0.53				
	DCR	0.99	0.94	1.04	0.66				
GPS	Three-month mortality	1.82	0.91	3.81	0.10				
	DCR	1.75	1.10	2.82	0.02	1.75	1.10	2.81	0.02
PNI	Three-month mortality	0.88	0.80	0.96	0.01	0.83	0.78	0.89	<0.001
	DCR	0.93	0.87	0.99	0.02	0.93	0.87	0.99	0.02
ALI	Three-month mortality	0.96	0.93	0.99	0.02	0.97	0.94	0.99	0.01
	DCR	0.98	0.97	1.00	0.04	0.99	0.97	1.00	0.03
Age	Three-month mortality	1.00	0.96	1.03	0.88	1.00	0.96	1.03	0.90
	DCR	0.99	0.97	1.02	0.65	0.99	0.97	1.02	0.66
Sex	Three-month mortality	1.04	0.57	1.89	0.90	0.99	0.54	1.78	0.96
	DCR	0.93	0.60	1.45	0.75	0.94	0.60	1.46	0.77
Clinical stage	Three-month mortality	0.95	0.36	2.89	0.93	1.05	0.41	3.13	0.92
	DCR	2.16	1.07	4.58	0.04	2.15	1.07	4.56	0.04
Monotherapy	Three-month mortality	1.82	0.78	4.66	0.18	1.97	0.86	5.00	0.13
	DCR	2.28	1.22	4.40	0.01	2.29	1.23	4.43	0.01
Treatment line 2	Three-month mortality	0.68	0.34	1.37	0.27	0.72	0.36	1.44	0.34
	DCR	1.32	0.79	2.21	0.29	1.33	0.80	2.23	0.27
Treatment line 3	Three-month mortality	1.42	0.57	3.46	0.44	1.49	0.60	3.59	0.38
	DCR	1.19	0.56	2.51	0.65	1.20	0.56	2.53	0.63
Comorbidities	Three-month mortality	1.03	0.54	2.01	0.93	0.98	0.52	1.91	0.95
	DCR	0.89	0.55	1.45	0.63	0.89	0.55	1.45	0.64

Abbreviations: NLR = Neutrophil to Lymphocyte Ratio (absolute count × 10^9^/L); GPS = Glasgow Prognostic Score, Glasgow Prognostic Score 0 = CRP ≤ 10 (mg/L) and albumin ≥ 35 (g/L), Glasgow Prognostic Score 1 = CRP > 10 (mg/L) and albumin ≥ 35 (g/L) or CRP ≤ 10 (mg/L) and albumin < 35 (g/L), Glasgow Prognostic Score 2 = CRP > 10 (mg/L) and albumin < 35 (g/L); CRP = C-Reactive Protein; PNI = Prognostic Nutrition Index = 10 × serum albumin value (g/dL) + 0.005 × total lymphocyte count/mm^3^; ALI = Advanced Lung Cancer Inflammation Index = BMI (kg/m^2^) × albumin (g/dL)/NLR; BMI = Body Mass Index.

**Table 5:** Bootstrapped Multivariable Logistic Regression Model for the Association between Nutritional and Inflammatory Biomarkers and Indexes, and Three-Month Mortality and Disease Progression in Patients with Non-Small Cell Lung Cancer Treated with Immune Checkpoint Inhibitors.

	Three-Month Mortality			Three-Month DCR		
Biomarker	OR	95% LCI	95% UCI	OR	95% LCI	95% UCI
GPS				1.79	1.10	2.99
PNI	0.83	0.76	0.89	0.92	0.86	0.99
ALI	0.96	0.94	0.99	0.99	0.97	1.00
Age	1.00	0.96	1.04	0.99	0.97	1.02
Sex	0.98	0.52	1.77	0.93	0.61	1.46
Clinical stage	1.13	0.43	3.78	2.18	1.09	4.56
Monotherapy	2.07	0.79	6.86	2.35	1.22	4.77
Treatment line 2	0.72	0.36	1.56	1.33	0.80	2.28
Treatment line 3	1.50	0.48	4.10	1.19	0.56	2.52
Comorbidities	1.00	0.54	2.00	0.88	0.56	1.48

Abbreviations: GPS = Glasgow Prognostic Score, Glasgow Prognostic Score 0 = CRP ≤ 10 (mg/L) and albumin ≥ 35 (g/L), Glasgow Prognostic Score 1 = CRP > 10 (mg/L) and albumin ≥ 35 (g/L) or CRP ≤ 10 (mg/L) and albumin < 35 (g/L), Glasgow Prognostic Score 2 = CRP > 10 (mg/L) and albumin < 35 (g/L); CRP = C-Reactive Protein PNI = Prognostic Nutrition Index = 10 × serum albumin value (g/dL) + 0.005 × total lymphocyte count/mm^3^; ALI = Advanced Lung Cancer Inflammation Index = BMI (kg/m^2^) × albumin (g/dL)/NLR; BMI = Body Mass Index.

## Data Availability

The datasets used for this study are available from the corresponding author upon reasonable request.

## Supplementary Materials

The following supporting information can be downloaded at: https://www.mdpi.com/article/10.3390/cancers18142185/s1. Table S1: Calculation of the nutritional and inflammatory biomarkers and indexes; Table S2: Baseline biomarker values stratified by treatment line; Figure S1: Receiver Operating Characteristic (ROC) Curve of Nutritional and Inflammatory Biomarkers for Predicting Three-Month Mortality in Patients with Non-Small Cell Lung Cancer Treated with Immune Checkpoint Inhibitors; Figure S2: Receiver Operating Characteristic (ROC) Curve of Nutritional and Inflammatory Biomarkers for Predicting Disease Progression in Patients with Non-Small Cell Lung Cancer Treated with Immune Checkpoint Inhibitors; Table S3: Adjusted Generalized Variance Inflation Factors (GVIF) for Biomarker Predictors; Table S4: Multivariable Logistic Regression Analysis for the Sensitivity Analysis of Disease Progression in Patients with Non-Small Cell Lung Cancer Treated with Immune Checkpoint Inhibitors; Figure S3: Receiver Operating Characteristic (ROC) Curve for Sensitivity Analysis with a RECIST-Based 3-Month Progression Endpoint in Patients with Non-Small Cell Lung Cancer Treated with Immune Checkpoint Inhibitors.

## Abbreviations

The following abbreviations are used in this manuscript:

ICI	Immune checkpoint inhibitor
NSCLC	Non-small cell lung cancer
CRP	C-Reactive Protein
NLR	Neutrophil-to-Lymphocyte ratio
GPS	Glasgow Prognostic Score
PNI	Prognostic Nutrition Index
ALI	Advanced Lung Cancer Inflammation Index
ORR	Objective response rate
OS	Overall survival
PFS	Progression-free survival
CR	Complete response
PR	Partial response
SD	Stable disease
PD	Progressive disease
AUC	Area under the curve
ROC	Receiver operating characteristic curve
