# Supplementary material for: The Association and Predictive Value of Nutritional and Inflammatory Biomarkers in Advanced Non-Small Cell Lung Cancer Response to Immune Checkpoint Inhibitors
Source: Cancers (Basel). 2026 Jul 8;18(14):2185. doi: 10.3390/cancers18142185 (PMC13407128; doi:10.3390/cancers18142185)

Supplementary materials

Table S1. Calculation of the nutritional and inflammatory biomarkers and indexes

**Biomarker**	**Calculation**
**NLR** [13]	Absolute neutrophil count * 10^9 /L/ absolute lymphocyte count * 10^9 /L
**GPS** [16]	• CRP > 10 (mg/L) and albumin < 35 (g/L) à 2 points<br>• CRP > 10 (mg/L) and albumin ≥ 35 (g/L) à 1 point<br>• CRP ≤ 10 (mg/L) and albumin < 35 (g/L) à 1 point<br>• CRP ≤ 10 (mg/L) and albumin ≥ 35 (g/L) à 0 points
**PNI** [14]	10 x serum albumin value (g/dL) + 0.005 x total lymphocyte count/mm^3
**ALI** [15]	BMI (kg/m^2) x albumin (g/dL) / NLR

Abbreviations: NLR = Neutrophil to Lymphocyte Ratio; CRP= C-Reactive Protein; GPS = Glasgow Prognostic Score; PNI = Prognostic Nutrition Index, ALI = Advanced Lung Cancer Inflammation Index

Table S2. Baseline biomarker values stratified by treatment line

		Treatment line			
**Baseline biomarker**	**Overall** N = 505[a]	**1st line** N = 177[a]	**2nd line** N = 276[a]	**3rd and further lines** N = 52[a]	**p-value**[b]
**CRP**	19.0 (7.0–62.0)	13.0 (6.0–48.0)	23.0 (9.0–68.0)	36.0 (10.0–62.0)	0.01
**Albumin**	40.5 (37.0–43.0)	41.0 (38.0–44.0)	40.00 (37.0–43.0)	40.0 (36.0–42.0)	0.03
**NLR**	4.6 (3.2–7.4)	4.7 (3.2–8.3)	4.5 (3.1–6.7)	5.0 (3.8–10.0)	0.09
**GPS**					0.03
0	161 (33.2%)	70 (42.2%)	77 (28.5%)	14 (28.6%)	
1	269 (55.5%)	84 (50.6%)	156 (57.8%)	29 (59.2%)	
2	55 (11.3%)	12 (7.2%)	37 (13.7%)	6 (12.2%)	
**PNI**	41.0 (38.0–43.0)	41.0 (38.0–44.0)	40.0 (38.0–43.0)	40.0 (36.0–42.0)	0.04
**ALI**	22.1 (12.9–33.3)	21.69 (12.3–33.3)	23.62 (14.1–34.2)	19.15 (10.3–27.5)	0.11
[a] Median (Q1–Q3); n (%)					
[b] Kruskal-Wallis rank sum test; Pearson's Chi-squared test					

Abbreviations: CRP= C-Reactive Protein; NLR = Neutrophil to Lymphocyte Ratio; GPS = Glasgow Prognostic Score; PNI = Prognostic Nutrition Index, ALI = Advanced Lung Cancer Inflammation Index

Figure S1. Receiver Operating Characteristic (ROC) Curve of Nutritional and Inflammatory Biomarkers for Predicting Three-Month Mortality in Patients with Non-Small Cell Lung Cancer Treated with Immune Checkpoint Inhibitors

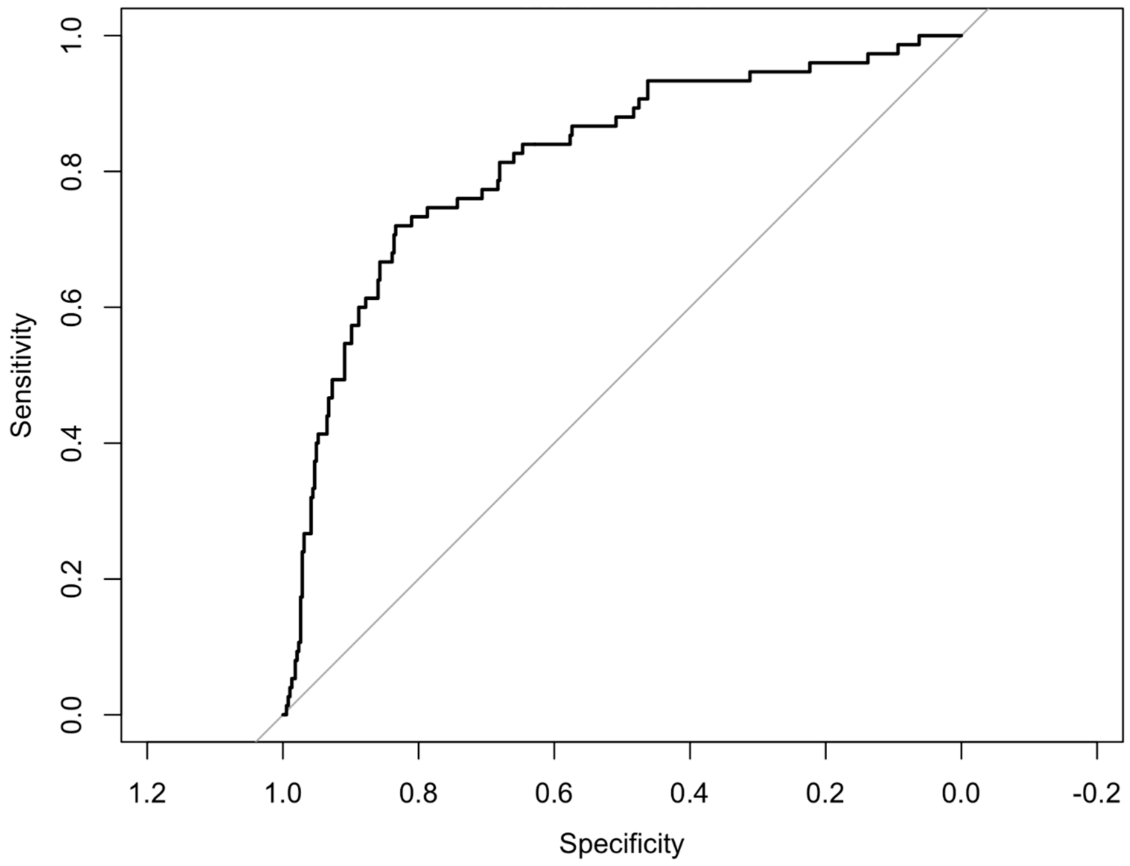

Figure S2. Receiver Operating Characteristic (ROC) Curve of Nutritional and Inflammatory Biomarkers for Predicting Disease Progression in Patients with Non-Small Cell Lung Cancer Treated with Immune Checkpoint Inhibitors

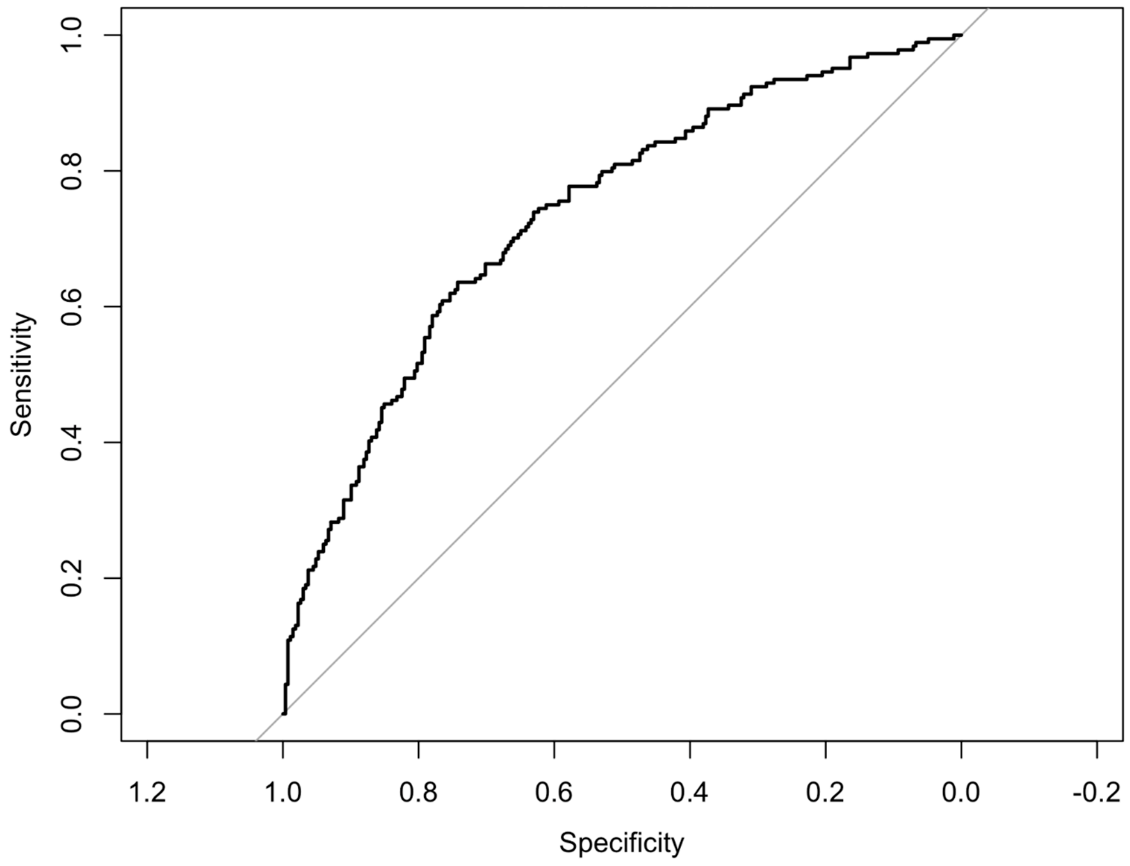

Table S3. Adjusted Generalized Variance Inflation Factors (GVIF) for Biomarker Predictors

Predictors	Adjusted GVIF
**3 -months mortality model**	
**PNI**	1.10
**ALI**	1.10
**Age**	1.11
**Sex**	1.04
**Clinical stage**	1.03
**Monotherapy**	1.06
**Treatment line**	1.05
**Comorbidities**[a]	1.08
**3-months progression model**	
**GPS**	1.321
**PNI**	1.33
**ALI**	1.10
**Age**	1.14
**Sex**	1.05
**Clinical stage**	1.03
**Monotherapy**	1.07
**Treatment line**	1.05
**Comorbidities**[a]	1.09

Abbreviations: GPS = Glasgow Prognostic Score; PNI = Prognostic Nutrition Index, ALI = Advanced Lung Cancer Inflammation Index

a. Included diabetes, hypertension, COPD, rheumatological conditions, dementia and cardiovascular diseases

Table S4. Multivariable Logistic Regression Analysis for the Sensitivity Analysis Disease Progression in Patients with Non-Small Cell Lung Cancer Treated with Immune Checkpoint Inhibitors

Variable	OR	95% LCI	95% UCI	p-value
**GPS**	1.67	1.05	2.66	0.03
**PNI**	0.93	0.87	0.99	0.02
**ALI**	0.99	0.97	1.00	0.02
**Age**	1.00	0.97	1.02	0.68
**Sex**	0.94	0.61	1.46	0.79
**Clinical stage**	2.22	1.11	4.70	0.03
**Monotherapy**	2.10	1.14	4.00	0.02
**Treatment line 2**	1.45	0.87	2.42	0.15
**Treatment line 3**	1.18	0.56	2.47	0.66
**Comorbidities**[a]	0.96	0.60	1.56	0.87

Abbreviations: GPS = Glasgow Prognostic Score; PNI = Prognostic Nutrition Index, ALI = Advanced Lung Cancer Inflammation Index

a. Included diabetes, hypertension, COPD, rheumatological conditions, dementia and cardiovascular diseases

Figure S3. Receiver Operating Characteristic (ROC) Curve for Sensitivity Analysis with a RECIST-Based 3-Month Progression Endpoint in Patients with Non-Small Cell Lung Cancer Treated with Immune Checkpoint Inhibitors

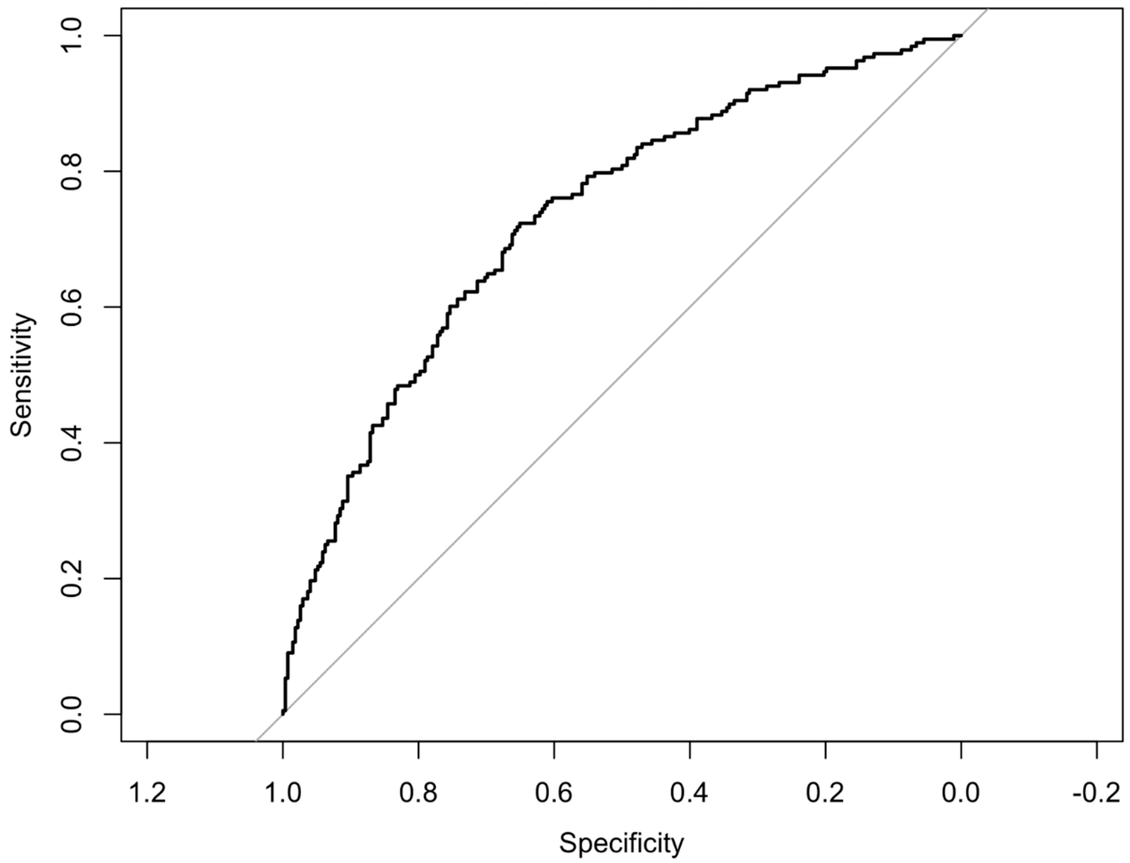

Supplement: Supplementary file 1 [file cancers-18-02185-s001.zip › cancers-4372661-supplementary.pdf]
